# African animal trypanosomosis (nagana) in northern KwaZulu-Natal, South Africa: Strategic treatment of cattle on a farm in endemic area

**DOI:** 10.4102/ojvr.v86i1.1639

**Published:** 2019-05-30

**Authors:** Abdalla A. Latif, Lundi Ntantiso, Chantel de Beer

**Affiliations:** 1 School of Life Sciences, University of KwaZulu-Natal, Westville, Durban, South Africa; 2 Makhathini Research Station, Jozini, South Africa; 3 Agricultural Research Council-Onderstepoort Veterinary Research, Pretoria, South Africa

**Keywords:** tsetse flies, trypanosomes, nagana, trypanocides treatment, KwaZulu-Natal

## Abstract

African animal trypanosomosis (AAT) is caused by several species of the genus *Trypanosoma*, a parasitic protozoan infecting domestic and wild animals. One of the major effects of infection with pathogenic trypanosome is anaemia. Currently, the control policies for tsetse and trypanosomosis are less effective in South Africa. The only response was to block treat all infected herds and change the dip chemical to one which controls tsetse flies during severe outbreaks. This policy proved to be less effective as demonstrated by the current high level of trypanosome infections in cattle. Our objective was to study the impacts of AAT (nagana) on animal productivity by monitoring the health of cattle herds kept in tsetse and trypanosomosis endemic areas before and after an intervention that reduces the incidence of the disease. The study was conducted on a farm in northern KwaZulu-Natal which kept a commercial cattle herd. There was no history of any cattle treatment for trypanosome. All cattle were generally in poor health condition at the start of the study though the herd received regular anthelminthic treatment. A treatment strategy using two drugs, homidium bromide (ethidium) and homidium chloride (novidium), was implemented. Cattle were monitored regularly for 13 months for herd trypanosomosis prevalence (HP), herd average packed cell volume (H-PCV) and the percentage of the herd that was anaemic (HA). A total of six odour-baited H-traps were deployed where cattle grazed from January 2006 to August 2007 to monitor the tsetse population. *Glossina brevipalpis* Newstead and *Glossina austeni* Newstead were collected continuously for the entire study period. High trypanosomes HP (44%), low average H-PCV (29.5) and HA (24%) were rerecorded in the baseline survey. All cattle in the herd received their first treatment with ethidium bromide. Regular monthly sampling of cattle for the next 142 days showed a decline in HP of 2.2% – 2.8%. However, an HP of 20% was recorded by day 220 and the herd received the second treatment using novidium chloride. The HP dropped to 0.0% and HA to 0.0% by day 116 after the second treatment. The cow group was treated again by day 160 when the HP and HA were 27.3% and 11%, respectively. The same strategy was applied to the other two groups of weaners and the calves at the time when their HP reached 20%. Ethidium and novidium treatment protected cattle, that were under continuous tsetse and trypanosomosis challenge, for up to 6 months. Two to three treatments per year may be sufficient for extended protection. However, this strategy would need to be included into an integrated pest management approach combining vector control for it to be sustainable.

## Introduction

African animal trypanosomosis (AAT) is caused by several species of the genus *Trypanosoma*, a parasitic protozoan infecting blood and tissues of the host animal (Leak 1998). Trypanosomes are transmitted by several vector species of blood sucking flies of the genus *Glossina*, commonly known as tsetse flies. The tsetse belt in South Africa is limited to north-eastern KwaZulu-Natal occupying 12,000 km² and is conserved mainly in nature reserves, national and private game parks and resorts (De Beer et al. 2016; Kappmeier Green, Potgieter & Vreysen 2007). Other man-made habitats such as exotic timber forests and patches of evergreen indigenous forests found around river beds are also suitable habitat for the tsetse flies. The habitat has been extended when most commercial cattle farming areas have moved into game farming areas and resorts, thus allowing bushes, trees and vegetation to grow, creating more suitable tsetse breeding sites. De Beer et al. (2016) reported a greater extension of *Glossina brevipalpis* than was previously known. Currently, two species of tsetse species coexist, *Glossina brevipalpis* Newstead and *Glossina austeni* Newstead. The two species have different behavioural activities and *G. austeni* has been proven to be a more competent vector of trypanosome parasites (Motloang et al. 2012). Dispersal and re-invasion of the flies is continuous between different habitats (Esterhuizen et al. 2005). There is a wealth of entomological information on the ecology of the two tsetse species covering geographical distribution, development of odour-baited traps and control methods since 1990 (De Beer et al. 2016; Hendrickx 2002, 2007; Hendrickx et al. 2003; Kappmeier 2000; Kappmeier & Nevill 1999a, 1999b; Kappmeier, Nevill & Bagnall 1998; Kappmeier Green 2003). The limited grazing, small areas and degraded communal land force cattle to move to such tsetse habitat, exposing them to nagana challenge.

One of the major effects of infection with pathogenic trypanosome is anaemia (Leak 1998). The disease varies from acute to chronic forms. The acute form occurs soon after the infection, characterised by high parasitaemia and rapid fall of packed cell volume (PCV). The extent of the acute and chronic forms of the disease is determined by a number of factors: complete tolerance (no-illness) in the case of game animals; the virulence of the *Trypanosoma* species, for example, *Trypanosoma congolense*; and its level of parasitaemia (Connor & Van Den Bossche 2004; Murray & Gray 1984). The chronic stage that is typical in indigenous breeds can persist for an extended period in which case the affected animals lose condition and become increasingly anaemic and lethargic (Itty 1996). Marcotty et al. (2008) evaluated PCV values as an ‘indicator of trypanosomosis infections in cattle’ in tsetse infested areas.

Three different approaches are to be considered in studies of the impacts of AAT on animal productivity (Swallow 1999). The first approach involves longitudinal monitoring of trypanosome prevalence in cattle, their health and productivity in areas of known trypanosomosis risk. The second approach is to monitor the health and productivity of cattle herds kept in nearby areas of lower and higher levels of trypanosomosis risk during the same period of time. These two approaches have advantages in that the productivity indicators (herd average PCV and herd percentage anaemia) are measured for entire herds rather than for individual animals. These approaches have been adopted in AAT studies conducted in KwaZulu-Natal. Trypanosome infections in cattle were recorded at 21 of the 25 communal dip tanks which clearly showed that the disease was still abundant and highly prevalent in north-eastern KwaZulu-Natal (De Beer et al. 2016; Ntantiso et al. 2014).

The third approach is to monitor the health and productivity of cattle herds before and after an intervention that reduces the incidence of trypanosomosis. This approach was one of the objectives of this study by following the parasitaemia and associated anaemia in a cattle herd. Secondly, the study also aimed at investigating and appraising a control strategy based on herd treatment against trypanosomosis over time. Currently, the control and management policies for tsetse and nagana are less effective involving the change of tick dipping chemicals to one which controls both ticks and tsetse such as pyrethroids formulations. The last mass cattle treatment with the trypanocide drug was reported in 1990 after a serious nagana outbreak. During this outbreak, about 10 000 cattle died of nagana and over 100 000 were treated using ethidium bromide (Kappmeier et al. 1998). That once-off intervention was shown to be unsustainable over the years, and high levels of trypanosome infections in cattle is still documented (Ntantiso et al. 2014; Van den Bossche et al. 2006).

## Materials and methods

### Boomerang Farm

Boomerang Farm is mainly a sugar cane plantation; however, a commercial cattle herd is kept there. It is situated next to the Nhlozi Gate in the western shores section of the Isimangaliso Wetland Park. It has 180 head of cattle with an output of about 30 cattle sold per year. Cattle were grazed into 300 hectares of indigenous forest during dry seasons. They were also supplemented by the cane sugar residue after the harvest. There was no history of any cattle treatment for trypanosome in recent years. All cattle were generally in poor health condition at the start of the study though the herd received regular anthelminthic treatment.

### Tsetse population monitoring

A total of six odour-baited H-traps (Kappmeier 2000; Kappmeier & Nevill 1999b) were deployed on different sites where cattle grazed from January 2006 to August 2007. The traps were baited with odours to enhance trapping of *G. brevipalpis* (Kappmeier & Nevill 1999a). These baits consisted of 1-octen-3-ol and 4-methylphenol at a ratio of 1:8 that were released at 4.4 mg/hour and 7.6 mg/hour, respectively. The chemicals were dispensed from seven heat-sealed sachets (7 cm × 9 cm) made of low-density polyethylene sleeves (wall thickness 150 microns) placed near the entrance of the trap. A 300-mL brown glass bottle that dispensed acetone through a 6-mm hole in the lid at a rate of 350mg/hour was placed next to the H trap (Kappmeier Green 2002). Flies were collected in a 20% ethanol solution to which an antiseptic, Savlon^®^ (Johnson & Johnson, Pharmedica Laboratories (Pty) Ltd. Rattray Road, East London, South Africa) (0.4 mL/L) and formalin (0.4 mL/L) had been added to preserve the sampled flies as well as to combat ant and spider predation. Traps were emptied and serviced every 14 days. The number of each species collected over this period was counted and results expressed as apparent density (AD), that is, the number of flies per trap per day.

### Strategic use of trypanocides

A treatment strategy using two drugs, homidium bromide (phenanthridinium bromide salt, ethidium – CAMCO Animal Health, United Kingdom) at a dose rate of 1.0 mg/kg and homidium chloride (phenanthridinium chloride salt, novidium – Rhone-Merieux, France) at a dose rate of 1.0 mg/kg, was attempted which would allow the herd of cattle to thrive in the high tsetse and trypanosomiasis challenge area. Both drugs were administered intramuscularly, and both have therapeutic and prophylactic actions. Ethidium was only used in the first cow treatment as an emergency, while treatment using novidium followed throughout the study period. Novidium was not registered in South Africa but a special import permit was granted by the Department of Agriculture, Forestry and Fisheries (DAFF). The adult cattle were treated and monitored for 13 months, while the calves born in 2005 and 2006 were treated and monitored for 8 months and 3 months, respectively. The herd average packed cell volume (H-PCV), the percentage of cattle with trypanosome infection referred to as the herd prevalence (HP) and the percentage of cattle in a herd with PCV of 24% or less referred to as herd anaemia (HA) (Ntantiso et al. 2014; Van den Bossche & Rowlands 2001) were all calculated. The HP, H-PCV and HA, which give a good indication of the health status of the herd (Trail et al. 1991), were obtained from the three groups of cattle, that is, cows, weaners 2005 and calves 2006. The threshold for the treatment was decided arbitrarily to be HP of 20% which was noticed to produce HA of around 25%.

### Sample processing and examination

Blood was collected from the tail or jugular veins using 10 ml vacutainer tubes coated with ethylenediamine tetraacetic acid as anticoagulant. Sample processing was done on the site. Blood from each sample was decanted into plain microhaematocrit capillary tubes that were sealed with cristseal and centrifuged for 5 min at 9000 revolution per minute (rpm). After centrifugation, the PCV was determined. Animals with a PCV of 24% or less were considered anaemic (Murray & Dexter 1988; Ntantiso et al. 2014; Van den Bossche & Rowlands 2001). The buffy coat of each sample was extruded onto a microscope slide, covered with a cover slip and examined for motile trypanosomes under a compound microscope using ×40 magnification.

*Trypanosoma congolense* is the dominant species infecting cattle and buffalo in northern KwaZulu-Natal including the study farm (Mamabolo et al. 2009; Motloang et al. 2012; Van den Bossche et al. 2006). *Trypanosoma brucei* was never detected in these reports, while *Trypanosoma vivax* was not detected in cattle samples obtained from the study farm (Boomerang Farm) using molecular technique (Mamabolo et al. 2009). Therefore, *Trypanosoma* infection in cattle, that is, the HP, in this study refers to infections with *T. congolense*.

### Ethical considerations

Ethical approval for the study was obtained from the ^3^Agricultural Research Council – Animal Ethics Committee of the Onderstepoort Veterinary Institute (ref. 07/20/C174). The research project was funded and approved by the Department of Agriculture, Forestry and Fisheries (DAFF) (Project number: OV21/13/C142: Epidemiology of Animal Trypanosomosis in KwaZulu-Natal, South Africa).

## Results

### Tsetse population

**FIGURE 1:**
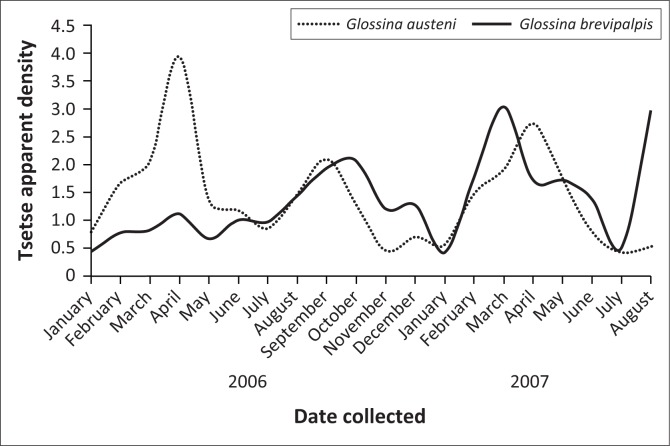
Monthly apparent density for *Glossina austeni* and *Glossina brevipalpis* at Boomerang Farm from January 2006 to August 2007.

A total number of 3892 *G. austeni* and 4107 *G. brevipalpis* were collected over the 16-month period with the six odour-baited H-traps, and there was no tsetse free period (Figure 1). The male to female ratio collected for *G. austeni* was 1:3.8 and for *G. brevipalpis* was 1:1.2. The monthly AD for *G. austeni* ranged from 3.9 in May 2006 at the end of the summer season to 0.4 in July 2007 in the middle of the winter season. The monthly AD for *G. brevipalpis* was the highest at 3.0 in March 2007, the middle of the summer season, and similar to *G. austeni* the lowest at 0.4 in July 2007. The average apparent densities for the collection period were similar for *G. austeni* (1.4 ± 0.9) and *G. brevipalpis* (1.4 ± 0.7). Three peaks in fly numbers were observed within the collection period for *G. austeni* (April 2006, September 2006 and April 2007) as well as for *G. brevipalpis* (October 2006, March 2007 and August 2007) (Figure 1).

### Strategic treatment of adult cattle and weaned calves at Boomerang Farm using trypanocidal drugs

**FIGURE 2:**
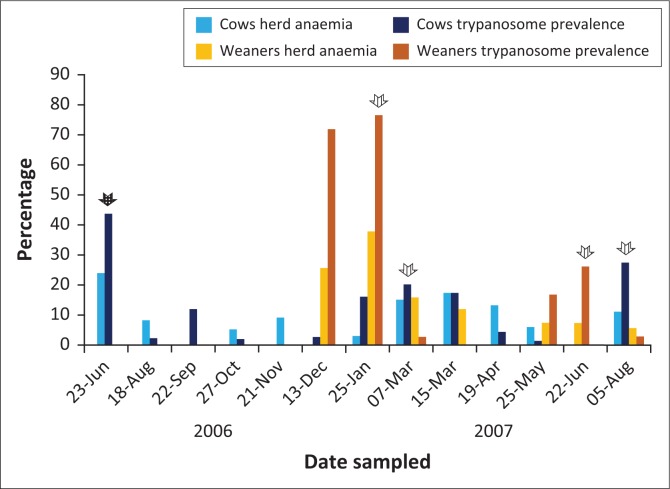
Trypanosome prevalence (%) and herd anaemia (%) for cows, and weaners at Boomerang Farm from 23 June 2006 to 05 August 2007. Dark arrow indicates ethidium treatment and striped arrow indicates novidium treatment of cattle.

Table 1 and Figure 2 show the results of HP, HA, H-PCV and timing of strategic treatment with ethidium bromide and novidium chloride. The primary survey conducted in June 2006 on the farm revealed very high trypanosomes HP (44%), H-PCV (29.5%) and HA (24%). Subsequently, all cattle in the cow herd received treatment with ethidium bromide (first treatment). Thereafter, the regular monthly sampling for the next 142 days showed a decline in HP of 2.2% – 2.8%. However, the HP of 16%, that is, five times the previous month, was recorded by day 185. This high level of infection was maintained for the following 2 months before the herd was treated by day 220 when the HP was 20% using novidium chloride. The HP and HA dropped to 0.0 and 0.0, respectively, by day 116 after the previous treatment. The cow group was treated again by day 160 after the previous treatment when the HP and HA were 27.3% and 11%, respectively.

**TABLE 1:** Herd average packed cell volume, trypanosome prevalence, herd anaemia and treatment strategy in cattle from June 2006 to August 2007 at Boomerang Farm.

Variable	Date sampled	Number of animals bled	Average PCV (± SD)	Herd anaemia (%)	Trypanosome prevalence (%)
**Cows**					
	23/06/2006	54	29.5 ± 6.7	24.0	44.0†
	18/08/2006	46	30.6 ± 4.5	8.0	2.2
	22/09/2006	34	33.4 ± 3.5	0.0	11.8
	27/10/2006	55	32.7 ± 4.4	5.0	1.9
	21/11/2006	34	33.0 ± 4.0	9.0	0.0
	13/12/2006	36	33.7 ± 3.9	0.0	2.8
	25/01/2007	40	32.4 ± 5.4	3.0	16,1
	01/03/2007	40	30.8 ± 5.2	15.0	20.0‡
	15/03/2007	35	30.1 ± 4.9	17.0	17.1
	19/04/2007	45	30.1 ± 5.1	13.0	4.4
	25/05/2007	77	30.8 ± 3.6	6.0	1.3
	22/06/2007	37	33.2 ± 4.8	0.0	0.0
	05/08/2007	66	30.2 ± 4.7	11.0	27.3‡
**Weaners (2005)**					
	13/12/2006	39	26.9 ± 3.5	28.2	71.8
	25/01/2007	42	26.4 ± 4.6	35.7	76.2‡
	01/03/2007	38	29.0 ± 3.7	15.8	2.6
	15/03/2007	34	27.7 ± 3.5	11.8	0.0
	19/04/2007	39	30.2 ± 3.1	0.0	0.0
	25/05/2007	42	28.3 ± 3.5	9.5	16.7
	22/06/2007	42	29.5 ± 3.7	4.7	26.2‡
	05/08/2007	36	30.3 ± 4.1	5,6	2.8
**Calves (2006)**					
	25/05/2007	27	35.3 ± 4.5	0.0	25.9
	22/06/2007	25	35.6 ± 3.7	0.0	20.0‡
	05/08/2007	26	33.3 ± 3.7	0.0	0.0

PCV, packed cell volume.

† , Treatment with ethidium bromide.

‡ , Treatment with novidium chloride.

In December 2006, at a time when the HP of the adult cows was very low (2.8%), the weaned calves (2005 group) experienced a very high HP of 71.8% while 25.6% of them were anaemic. This demonstrated the high trypanosomes challenge during this period. Forty-four days later, the HP of the group increased to 76% and the HA to 38.1%. All of the 2005 calves received treatment with novidium chloride (first treatment). The calves continued to be negative for trypanosomes infections with 0.0% HA for the following 80 days. This was followed by a second re-infection, and a high HP of 16.7% and 26.2% was recorded by day 116 and day 145, respectively, for the group treated with novidium chloride. This group remained with a very low HP (2.8%), with few cases being anaemic, HA was 5.6% for the rest of the observation period (43 days after the last treatment).

The 2006 calf group was again examined in May and June 2007 where the HP was 26% and 20%, respectively. The calves were treated using novidium and remained uninfected for the following 43 days when the observations on the farm cattle were terminated. The 2006 calf group did not show signs of anaemia during the investigation period.

Figure 2 shows the correlation between HP and HA; high and low HP before and after treatment correlated well with high and low HA.

## Discussion

Both *G. austeni* and *G. brevipalpis* had relatively high abundance throughout the study period. There were no tsetse free periods, and the cattle at Boomerang Farm were under constant vector pressure, which was higher in the hot or summer season. There was a positive association between these high tsetse apparent densities and infection prevalence in animals not treated with trypanocides. After treatment, this relationship did not exist, and it became apparent that these animals were protected against infection for up to 6 months.

Previous studies indicated that the vector competence of *G. austeni* for *T. congolense*, the most abundant *Trypanosoma* species in north-eastern KwaZulu-Natal, was significantly higher than that of *G. brevipalpis* (Motloang et al. 2012). However, cattle at dip tanks neighbouring a game park showed a similar high trypanosome prevalence in cattle where *G. austeni* was not recorded in the tsetse traps. The *Glossina brevipalpis* population was high through the years (Ntantiso et al. 2014) indicating that this species was most likely responsible for nagana transmission in cattle at these dip tanks. De Beer et al. (2016) proposed that the high trypanosome infection prevalence in cattle recorded in certain areas might be the result of the greater densities of *G. brevipalpis* relative to their lower vector competence.

The pathogenic *T. congolense* infecting cattle are responsible for the disease in KwaZulu-Natal (Mamabolo et al. 2009; Motloang et al. 2014; Van den Bossche et al. 2006). Different strains of *T. congolense* with great variation in their virulence have been reported (Bengaly et al. 2002; Masumu et al. 2006; Van den Bossche et al. 2011). Recently, two genetically distinct types of *T. congolense*, Savannah and Kilifi, have been isolated from cattle and tsetse flies in KwaZulu-Natal (Mamabolo et al. 2009; Motloang et al. 2014). Of the two genetically distinct types of *T. congolense* isolated from cattle and tsetse flies in KwaZulu-Natal, the Savannah sub-type is more prevalent and thought to be responsible for AAT outbreaks in cattle.

Motloang et al. (2014) reported the first attempt to determine the geographical distribution of virulent *T. congolense* strains in KwaZulu-Natal. Their results confirmed the higher virulence of the *T. congolense* Savannah type compared to the Kilifi type and indicated the prevalence of highly virulent strains to be higher in wildlife parks and in cattle near the parks than on farms further away. Ntantiso et al. (2014) carried out intensive and systematic studies on the epidemiology of cattle trypanosomosis from 2005 to 2008 (Ntantiso et al. 2014) in cattle neighbouring a game park. Over their study period comprising 1318 observations, they found that 62% of the trypanosome-infected cattle were anaemic, compared to 20.0% anaemia in the uninfected group. These results demonstrated the virulence of trypanosomes in cattle near the game parks.

The H-PCV recorded in Boomerang cattle were higher in infected cattle compared to infected cattle grazed near the game parks during the same observation period as judged by the smaller percentage of HA in the Boomerang cows group. Other than the less virulent *T. congolense* challenge in Boomerang cattle with reference to the findings by Motloang et al. (2014), cattle also received better grazing supplemented with sugar cane residues.

The first integrated tsetse and tick control was introduced in 1990, when a severe nagana outbreak occurred in the tsetse infested areas of north-eastern KwaZulu-Natal, about 10 000 cattle died of AAT and 116, 000 were treated using ethidium bromide during this outbreak (Kappmeier et al. 1998). The control measures included the use of pyrethroid dip, a chemical which has an insecticidal and acaricidal activity, on a 4-year interval to control the outbreak and challenge by the vector tsetse flies. This proposed management regime has not been followed, and 16 years following the 1990 outbreak, 76 cattle suspected to be infected with trypanosome were bled at one communal diptank at the edge of the Hluhluwe-uMfolozi Park and the results were reported in a research communication by Van den Bossche et al. (2006). Thirty-four per cent of cattle were found to be infected with *T. congolense* and 83% were anaemic. This once-off survey demonstrated that nagana was still prevalent and recommended further research to develop appropriate control methods.

The intention of this study was to treat adult cows and calves at an arbitrary HP threshold of 20% before the disease produces significant production losses. The PCV of individual animals and the H-PCV are useful indicators of anaemia, and in trypanosome, endemic areas are the most typical signs of nagana in domestic animals (Marcotty et al. 2008; Murray & Dexter 1988; Trail et al. 1991). Ethidium and novidium strategic treatment produced attractive results whereby cattle were protected for an extended period of up to 6 months. Therefore, two to three treatments per year may be sufficient to keep cattle productivity on the farm under all year tsetse and trypanosomosis challenge. It is noted that the trypanosomes HP and the consequent HA reached very high levels in 2005 born calves (76% and 37%, respectively). If this group had not been treated, the weaned calves could have experienced a state of ‘stunted growth’ and become unfit for sale. Additionally, calves, with reference to the group born in 2006, seemed to resist HP of up to 20% without showing recognised signs of anaemia (none of the calves were found with PCV equal or less than 24%). This observation proved the ‘arbitrary threshold for treatment’ adopted in this study. Age-related resistance to trypanosomes is recognised where anaemia in infected calves was moderate (Maclennan 1974; Murray, Morrison & Whitelaw 1982; Valli, Forsberg & McSherry 1978; Wellde et al. 1981) as well as young animals which are less attractive to tsetse flies. Ethidium and novidium were reported to give protection for a period of up to 4 months (Brander & Pugh 1977). There were some successes reported of farming in tsetse and trypanosomes challenge areas (Holmes & Scott 1982; Logan et al. 1984; Moloo et al. 1988; Trail et al. 1985) and in Zimbabwe and Mozambique (Boyt 1979; Takken, Taylor-Lewis & Woodford 1988). The strategic use of trypanocides requires close monitoring of the HP through veterinary supervision, surveillance and strict administration of the drugs (Connor & Van den Bossche 2004; Holmes & Scott 1982) to avoid under-dosing or overdosing of a drug which may shorten the time for the trypanosomes to build resistance against the drug. The problem of development of resistance in trypanosomes is the threat to the sustainability of the strategy. It is noteworthy that an investigation into drug-resistant strains in KwaZulu-Natal was carried out and the results did not reveal the presence of any resistant strains (Justin Masumu, pers. comm., September 2010). In the absence of a tsetse eradication policy, integrated approaches can be applied for the control of trypanosomosis (Holmes 1997; Murray & Black 1985) including animal treatment and tsetse fly suppression by using deltamethrin-treated cattle, targets and screens (Bauer et al. 2011; Hargrove, Torr & Kindness 2003; Torr, Maudlin & Vale 2007).

## Conclusion

The tsetse and trypanosomosis high challenge had been continuous over the years (Figure 1, Table 1). The strategic treatment using ethidium bromide and novidium chloride produced promising results whereby cattle were protected for extended period of up to 6 months. Therefore, two to three treatments per year may be sufficient to keep cattle productivity on the farm under tsetse and trypanosomosis continuous challenge. However, this strategy can be sustainable if an integrated management of tsetse and AAT is implemented by suppression of the tsetse fly challenge.
